# Exogenous Heat Shock Cognate Protein 70 Suppresses LPS-Induced Inflammation by Down-Regulating NF-κB through MAPK and MMP-2/-9 Pathways in Macrophages

**DOI:** 10.3390/molecules23092124

**Published:** 2018-08-23

**Authors:** Erna Sulistyowati, Mei-Yueh Lee, Lin-Chi Wu, Jong-Hau Hsu, Zen-Kong Dai, Bin-Nan Wu, Ming-Chung Lin, Jwu-Lai Yeh

**Affiliations:** 1 Graduate Institute of Medicine, College of Medicine, Kaohsiung Medical University, Kaohsiung 807, Taiwan; dr_erna@unisma.ac.id (E.S.); jhh936@yahoo.com.tw (J.-H.H.); zenkong@kmu.edu.tw (Z.-K.D.); binnan@kmu.edu.tw (B.-N.W.); 2 Faculty of Medicine, Islamic University of Malang, East Java 65145, Indonesia; 3 Division of Endocrinology and Metabolism, Department of Internal Medicine, Kaohsiung Medical University Hospital, Kaohsiung 807, Taiwan; lovellelee@hotmail.com; 4 Department of Internal Medicine, Kaohsiung Municipal Hsiaokang Hospital, Kaohsiung 812, Taiwan; 5 Department of Pharmacology, College of Medicine, Kaohsiung Medical University, Kaohsiung 807, Taiwan; a2813228@yahoo.com.tw; 6 Department of Pediatrics, College of Medicine, Kaohsiung Medical University, Kaohsiung 807, Taiwan; 7 Department of Pediatrics, Kaohsiung Medical University Hospital, Kaohsiung 807, Taiwan; 8 Department of Anesthesiology, Chi Mei Medical Center, Liouying, Tainan 736, Taiwan; 9 Department of Medical Research, Kaohsiung Medical University Hospital, Kaohsiung 807, Taiwan

**Keywords:** heat shock protein, lipopolysaccharide, inflammation, RAW264.7 macrophages, matrix metalloproteinases

## Abstract

Heat shock cognate protein 70 (HSC70), a molecular chaperone, is constitutively expressed by mammalian cells to regulate various cellular functions. It is associated with many diseases and is a potential therapeutic target. Although HSC70 also possesses an anti-inflammatory action, the mechanism of this action remains unclear. This current study aimed to assess the anti-inflammatory effects of HSC70 in murine macrophages RAW 264.7 exposed to lipopolysaccharides (LPS) and to explain its pathways. Mouse macrophages (RAW 264.7) in 0.1 µg/mL LPS incubation were pretreated with recombinant HSC70 (rHSC70) and different assays (Griess assay, enzyme-linked immune assay/ELISA, electrophoretic mobility shift assay/EMSA, gelatin zymography, and Western blotting) were performed to determine whether rHSC70 blocks pro-inflammatory mediators. The findings showed that rHSC70 attenuated the nitric oxide (NO) generation, tumor necrosis factor α (TNF-α) and interleukin 6 (IL-6) expressions in LPS-stimulated RAW264.7 cells. In addition, rHSC70 preconditioning suppressed the activities and expressions of matrix metalloproteinase-2 (MMP-2) and MMP-9. Finally, rHSC70 diminished the nuclear translocation of nuclear factor-κB (NF-κB) and reduced the phosphorylation of extracellular-signal regulated kinases 1/2 (ERK1/2), c-Jun N-terminal kinase (JNK), p38 mitogen-activated protein kinases (MAPK), and phosphatidylinositol-3-kinase (PI3K/Akt). We demonstrate that rHSC70 preconditioning exerts its anti-inflammatory effects through NO production constriction; TNF-α, and IL-6 suppression following down-regulation of inducible nitric oxide synthase (iNOS), cyclooxygenase 2 (COX-2), and MMP-2/MMP-9. Accordingly, it ameliorated the signal transduction of MAPKs, Akt/IκBα, and NF-κB pathways. Therefore, extracellular HSC70 plays a critical role in the innate immunity modulation and mechanisms of endogenous protective stimulation.

## 1. Introduction

HSC70 (73 kDa) is one of the four major members of the heat shock proteins (HSPs), which is a constitutively expressed molecular chaperone located in various cellular locations such as the nucleus and cytoplasm [1]. Human HSC70 has three basic structures, including a 44 kDa amino-terminal an adenosine triphosphatase (ATPase) domain (an ATP-binding domain), an 18 kDa peptide (substrate) binding domain, and a 10 kDa carboxyl-terminal domain [2,3,4]. HSC70 is a well-defined ATP binding chaperone and displays intrinsic ATPase activity which can hydrolyze ATP into ADP [5]. In a cell, HSC70 is abundantly expressed and comprises approximately 1% of the total protein. Although mainly localized in the cytoplasm, HSC70 has also been reported to be connected with endosomes, exosomes, and lysosomes [6]. In eukaryotic cells, HSC70 can transverse out of and return to the nucleus to and from the cytoplasm. Cellular stress such as heat shock or oxidative stress produced by H_2_O_2_ stimulate HSC70 accumulation in nuclei. Its nucleocytoplasmic shuttle is inhibited by stress and isolates in nuclei [7,8].

HSC70 has many essential functions. It maintains protein homeostasis (protein folding, translocation, assembly, disassembly, differentiation, degradation in both normal and stress stimulation [9], catalyzes ATP-dependent un-coating of clathrin-coated pits [10], partakes in the process of new protein synthesis [11], co-regulates cellular signaling and functions with other molecular chaperones [12] and other significant cellular activities. Interestingly, in the immune system, HSC70 modulates antigen transport within cells to control major histocompatibility complex (MHC) class II presentation during cellular stress [13].

Our recent study showed that in rats with septic shock, exogenous HSC70 can prevent LPS-induced cardiac and hepatic dysfunction [14]. These protection against endotoxemia-induced cardiac and hepatic dysfunctions are associated with anti-inflammatory effects, as revealed by the inhibition of proinflammatory mediators including TNF-α, NO, COX-2, and MMP-9 via the MAPK/NF-κB pathway. Exogenous mammalian HSP70 has been shown to protect against the harmful effects of bacterial pathogens, including lipoteichoic acid and LPS [15]. The in vivo and in vitro studies suggested that extracellular mammalian HSP70 possess important functions in the modulation of innate immunity and stimulation of endogenous protective mechanisms at both the cellular and organism level. HSP70 is present in cells under normal physiological conditions. The abnormal expression of HSC70 and anomalous HSC70 function may lead to various diseases. Certain studies have demonstrated that high levels of HSC70 are found after different cellular stresses, including inflammation, infection, and cancer [16,17]. Specifically in the immune system, HSP70 is highly immunogenic and is central in the modulation of endocytic and autophagy pathways resulting in antigens being presented to MHC class II. These MHC class II molecules are located in the surface of professional Ag presenting cells (APCs): dendritic cells, B cells, and macrophages as well as some endothelial, epithelial and tumor cells [18]. 

High exogenous addition of HSC70 may lead to various responses in many different types of cells. Incubation of human monocytes with HSP70 elicits a rapid intracellular calcium flux, activates NF-κB, and up-regulates the expression of pro-inflammatory cytokines [17]. On the other hand, HSP70 preconditioning decreases NO production in LPS-stimulated macrophages [19]. In addition, extracellular HSP70 modifies mononuclear cell responses to subsequent LPS challenge, and HSP70 preconditioning attenuates cytosolic degradation of inhibitor kappa B-alpha (IκB-α), inhibits activation of IκB kinase, and decreases phosphorylation of the p65 subunit of NF-κB following LPS stimulation [20]. HSC70 shares part of the structural and functional similarity with HSP70. However, HSC70 has its own pathways in the regulation of NF-κB-related inflammatory mediators such as iNOS, COX-2, MMP-9 and MAPK signaling pathway. Therefore, we evaluate whether high levels of exogenous HSC70 administration can affect one of the key players in the immune response, macrophages, through an in vitro study. The aims of this study were to determine the anti-inflammatory effects and to evaluate the mechanisms of HSC70 in murine macrophages (RAW 264.7) exposed to LPS. We tested the hypothesis that HSC70 preconditioning results in the down-regulation of iNOS, COX-2, and MMP-2/MMP-9 by inhibition of signal transduction through MAPK, Akt/IκB-α, and NF-κB pathways.

## 2. Results

### 2.1. Effects of rHSC70 on LPS-Induced iNOS and COX-2 Expression

**Figure 1:**
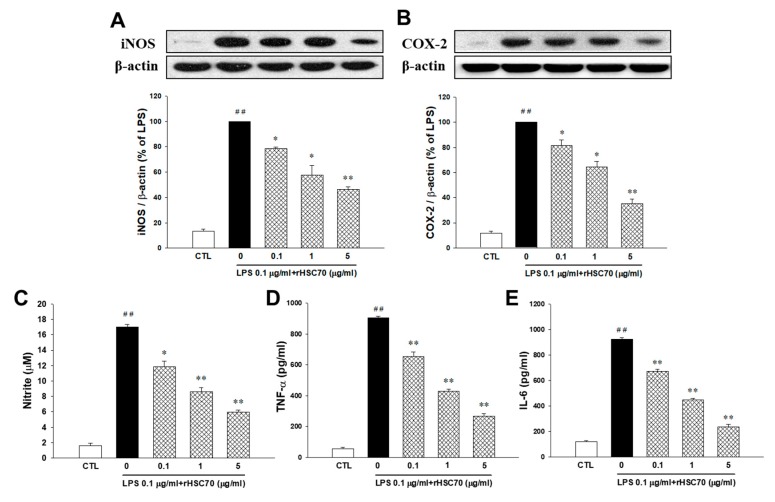
Effects of rHSC70 on LPS-induced iNOS (**A**) and COX-2 (**B**) expression, nitrite (**C**), TNF-α (**D**) and IL-6 (**E**) production in RAW264.7 cells. Cells were pretreated with recombinant heat shock cognate protein 70 (rHSC70; 0.1, 1 and 5 μg/mL) for 5 min before LPS treatment and protein samples were prepared 24 h after LPS treatment. Densitometric analysis showed that the relative expression level of iNOS and COX-2. The β-actin was used as a loading control. Significant differences were identified by One-Way ANOVA followed by Dunnett’s test. Each value represents as mean ± SEM of three independent experiments, with triplicate determinations in each experiment. ## *p* < 0.01 versus control group (CTL); * *p* < 0.05, ** *p* < 0.01 versus LPS group.

As depicted in Figure 1A, iNOS was upregulated following LPS treatment (0.1 µg/mL) alone and after pretreatment with rHSC70 at 0.1 and 1 µg/mL as a comparison with the control group, whereas, the LPS-induced upregulation of iNOS was attenuated in 5 µg/mL of rHSC70. As shown in Figure 1B, COX-2 was upregulated following LPS treatment (0.1 µg/mL) alone and pretreatment with rHSC70 at 0.1 µg/mL compared with the control group. Conversely, the LPS-stimulated upregulation of COX-2 was attenuated in the pretreatment with rHSC70 at 1 and 5 µg/mL as a comparison with the control group. 

### 2.2. Effects of rHSC70 on LPS-Induced Nitrite, TNF-α and IL-6 Production

As shown in Figure 1C,E, compared with the control group, nitrite, TNF-α, and IL-6 were upregulated following LPS treatment (0.1 µg/mL) and pretreatment with rHSC70 at 0.1, 1 and 5 µg/mL. However, in the LPS-induced upregulation of nitrite, TNF-α and IL-6 were attenuated in a dose-dependent manner.

### 2.3. Effects of rHSC70 on LPS-Induced Phosphorylation on IκBα, NF-κB p65 Translocation and NF-κB Transcription Factor Binding

**Figure 2:**
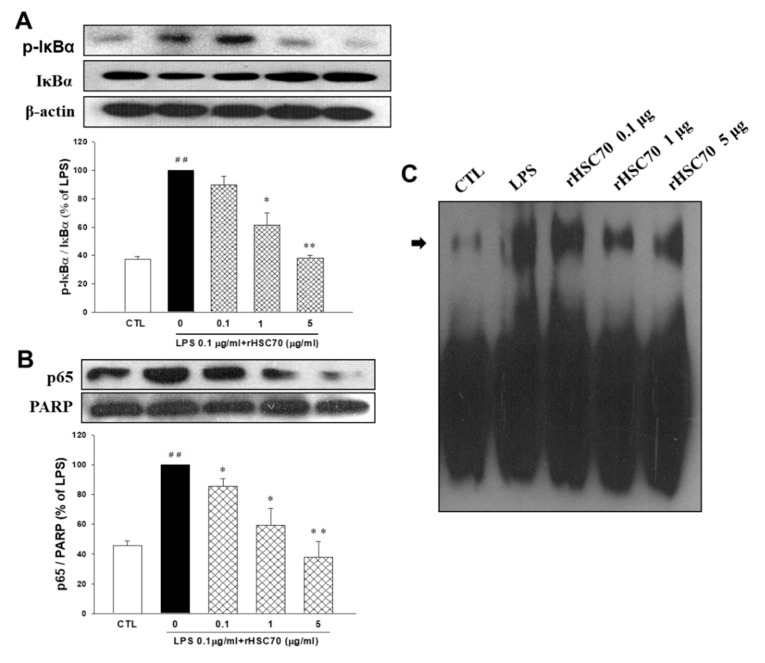
Effects of rHSC70 on LPS-induced phosphorylation of IκBα (**A**) and NF-kB translocation (**B**,**C**). Cells were pretreated with rHSC70 (0.1, 1 and 5 μg/mL) for 5 min before LPS treatment and protein samples were prepared 30 min after LPS treatment. (**A**) The cytosolic fractions were used to analyze the content of IκBα and phosphorylated IκBα. Western blotting shows that LPS induced IκBα phosphorylation was attenuated by rHSC70. (**B**) The nuclear fractions were used to analyze the content of NF-kB p65. Western blotting shows that rHSC70 attenuated LPS-induced NF-kB activation. (**C**) NF-κB binding was determined by EMSA. Significant differences were identified by One-Way ANOVA followed by Dunnett’s test. Each value represents as mean ± SEM of three independent experiments. ## *p* < 0.01 versus control group (CTL); * *p* < 0.05, ** *p* < 0.01 versus LPS group.

**Figure 3:**
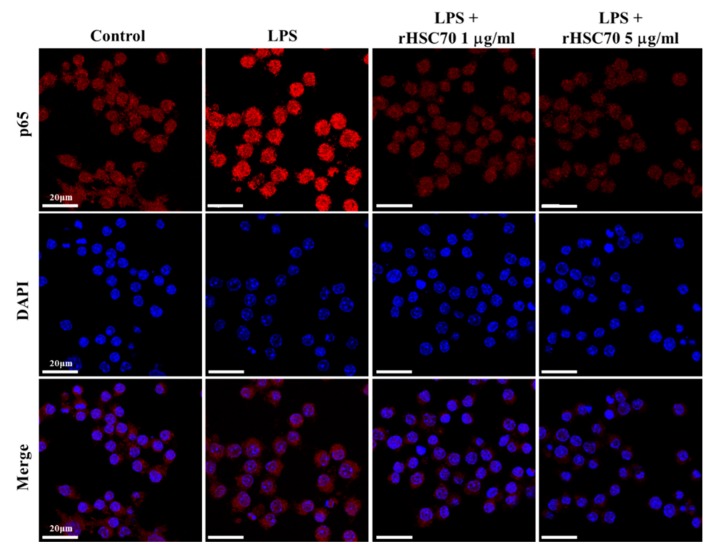
Effects of rHSC70 on LPS-induced translocation of NF-kB. Cells were pretreated with rHSC70 (1 and 5 μg/mL) for 5 min before LPS treatment and 30 min after LPS treatment, the cells followed by immunocytochemistry staining. For the counterstaining, rhodamine-labeled mouse antibody was used. The rHSC70 inhibited LPS-induced translocation of p65 as shown by the location of anti-p65 stain with the nucleus stained with DAPI. Scale bar: 20 µm.

Figure 2 shows that IκBα and NF-κB p65 were upregulated following LPS treatment (0.1 µg/mL) alone and pretreatment with rHSC70 at 0.1 and 1 µg/mL as a comparison with the control group. Aside from that, the LPS-induced upregulations of IκBα and NF-κB p65 were attenuated in dose-response relationship (Figure 2A,B). Compared to the LPS treatment group, the expressions of IκBα and NF-κB p65 were attenuated following pretreatment with 5 µg/mL rHSC70. Subsequently, nuclear extracts were made, and EMSA was used to determine the NF-κB binding. Similar results were obtained from three independent experiments (Figure 2C). However, as presented in Figure 3, pretreatment with rHSC70 at 1 and 5 µg/mL inhibited LPS-induced nuclear translocation of p65, a subunit of NF-κB.

### 2.4. Effects of rHSC70 on LPS-Induced the Activations of MMP-2 and MMP-9

**Figure 4:**
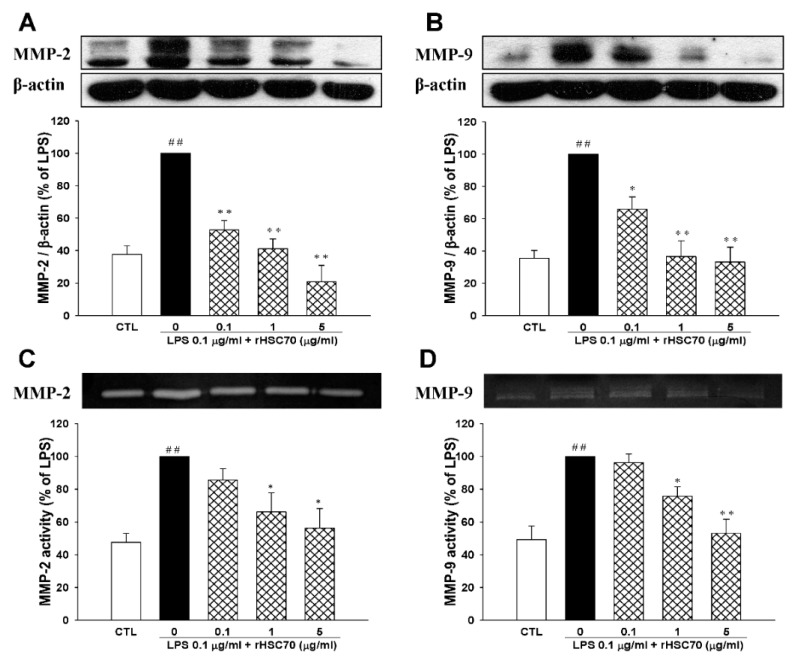
Effects of rHSC70 on LPS-induced activations of MMP-2 and MMP-9. RAW264.7 cells were pretreated with rHSC70 (0.1, 1 and 5 μg/mL) for 5 min before LPS treatment and protein samples were prepared 24 h after LPS treatment. (**A**,**B**) Effects on protein expressions of MMP-2 and MMP-9 were analyzed by western blotting. (**C**,**D**) Effects on activities of MMP-2 and MMP-9 were analyzed by gelatin zymography. Recombinant HSC70 inhibited LPS-induced MMP-2/-9 protein expression and its enzyme activities. Significant differences were identified by One-Way ANOVA followed by Dunnett’s test. Each value represents as mean ± SEM of three independent experiments. ## *p* < 0.01 versus control group (CTL); * *p* < 0.05, ** *p* < 0.01 versus LPS group.

Two important mediators of an inflammatory response in sepsis, MMP-2 and MMP-9 were determined in a dose-dependent manner. As shown in Figure 4A,B the protein expressions and activities of MMP-2 and MMP-9 were upregulated following LPS treatment (0.1 µg/mL) alone and pretreatment with rHSC70 at 0.1 and 1 µg/mL compared to the control group. Conversely, the LPS-induced upregulations of MMP-2 and MMP-9 were attenuated in a dose-dependent relationship as compared with control group. When compared with LPS treatment group, the expressions of MMP-2 and MMP-9 were also attenuated following pretreatment with 5 µg/mL rHSC70. In zymography assay (Figure 4C,D), we found that in the comparison with control group, MMP-2 and MMP-9 were upregulated following LPS treatment (0.1 µg/mL) alone and pretreatment with rHSC70 at 0.1, 1 and 5 µg/mL. On the contrary, the LPS-induced upregulations of MMP-2 and MMP-9 were attenuated in a dose-responsive manner.

### 2.5. Effects of rHSC70 on the Expressions of LPS-Induced Activations ERK1/2, JNK, p38 and Akt Pathways

**Figure 5:**
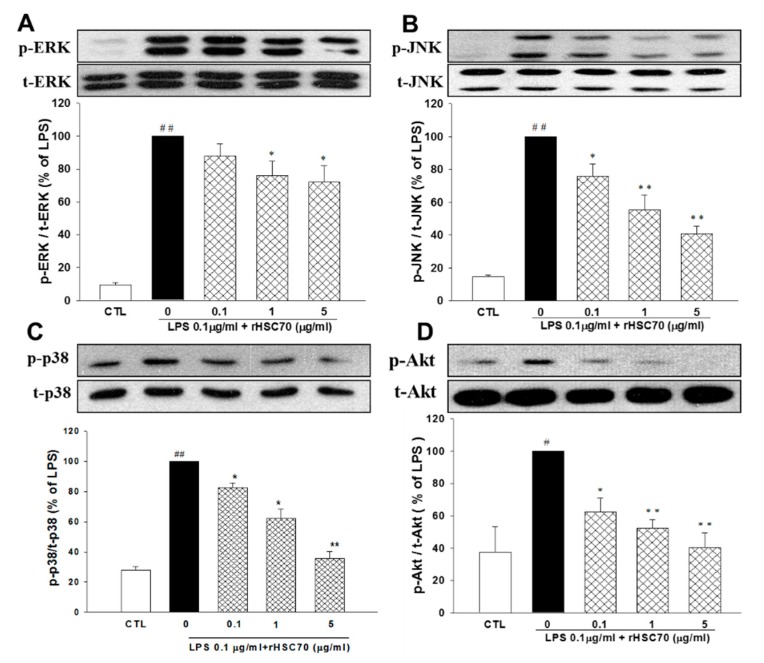
Effects of rHSC70 on LPS-induced activations of MAPKs and Akt pathways. Cells were pretreated with rHSC70 (0.1, 1 and 5 μg/mL) for 5 min before LPS treatment and protein samples were prepared 30 min after LPS treatment. Western blotting shows that rHSC70 attenuated LPS-induced phosphorylation of ERK 1/2 (**A**), JNK (**B**) and p38 (**C**) MAPK, and Akt (**D**). The total MAPK and Akt levels were used as internal controls. Significant differences were identified by One-Way ANOVA followed by Dunnett’s test. The results are reported as mean ± SEM of three independent experiments, with triplicate determinations in each experiment. # *p* < 0.05, ## *p* < 0.01 versus control group (CTL); * *p* < 0.05, ** *p* < 0.01 versus LPS group.

As presented in Figure 5, a western blotting assay was employed to determine the MAPK pathways including ERK1/2, JNK, p38 and Akt by already described method. In comparison with the control group, ERK1/2, JNK, p38, and Akt were upregulated following LPS treatment (0.1 µg/mL) alone and pretreatment with rHSC70 at 0.1, 1 and 5 µg/mL. On the contrary, the LPS-induced upregulations of ERK1/2, JNK, p38 and Akt were attenuated in a dose-dependent manner as compared with the control group.

## 3. Discussion

Our findings provide important evidence about extracellular HSC70 and macrophage stimulation. We found that exogenous HSC70 attenuated the generation of NO, TNF-α, and IL-6 from LPS-stimulated RAW 264.7 cells. In addition, HSC70 preconditioning attenuated the activity and protein expressions of MMP-2 and MMP-9. Additionally, HSC70 prohibited the nuclear translocation of NF-κB and suppressed the phosphorylation of ERK1/2, JNK, p38 MAPK, and Akt. Given the concerns about lack of effective treatments and the high rates of morbidity and mortality in patients with septic shock, extracellular HSC70 offers a promising therapeutic option to treat sepsis or forms the basis of a novel prophylactic strategy for the management of septic shock [21].

Patients with sepsis experience profound suppression of the innate immune system. The epithelial barriers and immune cells such as macrophages and dendritic cells express pathogen recognition receptors (PRRs) as a part of innate immune system, and function to detect the invasion of microorganisms [22]. TLRs are specific families of PRRs that recognize pathogen-associated molecular patterns (PAMPs), and is defined by conserved macromolecular motives from microorganisms, of which LPS is an example of bacterial PAMPs [23]. Stimulation of TLRs or the nucleotide-binding, oligomerization domain (NOD)-like receptor family of intracellular PRRs activates downstream signaling cascades which then leads to the activation of a transcriptional response program including NF-κB, followed by the generation of cytokines, chemokines, and NO [24,25]. Polymicrobial sepsis has been shown to result in the suppression of macrophage function through reducing the production of pro-inflammatory cytokines such as TNF-α, IL-1, IL-6, and IL-12 upon re-exposure to LPS in vitro [26]. Recent studies have attempted to identify unifying mechanisms through genome-wide expression data in the early and late stages of sepsis. However, inflammatory markers such as TNF-α, IL-1, and IL-10 have not revealed any consistent pattern with regards to gene expression, and have been shown to be highly variable from individual to individual. Taken together, these findings suggest that the host response to sepsis does not simply involve a pro-inflammatory phase followed by an anti-inflammatory response. Rather, it is an interactive and dynamic process which potentially involves heterogeneous genome-specific pathways.

TNF-α is the most frequently studied cytokine with regards to the pathophysiology of sepsis. TNF-α, a 17-kDa protein, predominantly derived not only from activated immune cell macrophages, but also from non-immune cells such as fibroblasts in response to invasive, infectious, and inflammatory stimuli [27]. Thirty min after stimulation, TNF-α was released from macrophages and followed gene transcription and RNA translation. Therefore, TNF-α is an important early regulator of the immune response. Injections of TNF-α into experimental animals have been shown to bear a resemblance to the syndrome of septic shock, and infusion of recombinant TNF-α into humans has been shown to result in systemic inflammatory response syndrome [28]. Importantly, TNF-α provides an important mediator for the development of fever, and thus can be classified as a pyrogenic cytokine [29,30]. Once released, TNF-α acts on target cells such as macrophages, neutrophils, and endothelial cells, which leading to enhance the generation of macrophages from progenitor cells [31]. Subsequently, it turn to promote the activation, differentiation and prolong survival of macrophages [32]. All of these effects enhance pro inflammatory responses in patients with sepsis. In addition, TNF-α amplifies the inflammatory cascades through autocrine and paracrine mechanisms due to macrophage-activated secretion of other pro inflammatory cytokines, such as IL-6, IL-8, and macrophage migration inhibitory factor, lipid mediators, and reactive oxygen and nitrogen species [33]. Subsequently, it leads to sepsis-induced organ dysfunction. Regarding the ability to affect downstream cytokine cascades, TNF-α has been described as a “master regulator” of inflammatory cytokine production [27].

IL-6 possesses a molecular mass of 21 kD. This glycoprotein is broadly produced by various types of cells like macrophages, lymphocytes, fibroblasts, and endothelial, dendritic and smooth muscle cells. These cells react in response to LPS, IL-1, and TNF-α stimulation [34]. Patients who suffer from burns, post-major surgery, and those with sepsis have been reported to have increased IL-6 levels [35], and the peak IL-6 level is associated with the concentration of TNF-α. In these conditions, the level of IL-6 in plasma is consistently high, and it is correlated with various indicators of disease severity including multi-organ failure and septic shock and global mortality [36]. Recent studies have been growing interest in the IL-6-knockout mice model, which the deletion of the IL-6 gene leads to prevent lung inflammation in an acute lung injury mouse model, and protects their mortality as well. Another study in zymosan-induced acute peritoneal inflammation demonstrated that IL-6 gene deletion inhibits the development of organ failure [37]. In the patients who undergo septic shock, myocardial dysfunction generates tissue perfusion damage, multi-organ failure, and mortality. Above all, this study demonstrated that LPS treatment led to upregulation of nitrites, TNF-α and IL-6, while pretreatment with rHSC70 at 0.1, 1 and 5 µg/mL attenuated their upregulation in a dose-dependent manner.

NO is produced primarily by the endothelium through constitutive isoform of NO synthase, which has an important effect on the regulation of blood pressure. Abundant studies suggested that in the patients with septic shock, iNOS-induced NO hyperproduction result in the hypotension, cardiac depression, and vascular hyporeactivity. Furthermore, LPS, TNF-α, IL-1 and IFN-γ have been found to stimulate iNOS in certain tissues such as endothelium, vascular smooth muscle cells, macrophages and parenchymal cells [38]. In the animal models, administration of NO synthesis inhibitors in patients with septic shock leads to improving hemodynamic status and perpetuation. The down-regulation of NO synthesis affects the hemodynamic condition in a short-term study of patients who suffer septic shock [39]. In the current study, we found that LPS treatment and rHSC70 pretreatment at 0.1 and 1 µg/mL caused iNOS upregulation. However, the LPS-induced upregulation of iNOS was attenuated with rHSC70 pretreatment at 5 µg/mL. COX-1 and COX-2 provide a fundamental role in inflammatory cascades by transforming arachidonic acid to prostaglandin H2, which then is converted to specific terminal synthases; bioactive prostanoids [40]. COX-2 possesses highly sensitive response to inflammatory stimuli, and thus it is potential to be the therapeutic target for anti-inflammatory medicine. In this current study, COX-2 was upregulated following LPS treatment and in pretreatment with rHSC70 at 0.1 µg/mL. Conversely, in the LPS-induced upregulation of COX-2 was constrained in pretreatment with rHSC70 at 1 and 5 µg/mL.

Commonly, NF-κB plays a pivotal role in sepsis, and it is characterized by its activation which is initiated by a signal from ubiquitylation of inhibitors of kappa B (IκBs). This signaling pathway is primarily through IκB kinase (IKK) activation, which subsequently leads its degradation and implicates in the multi-organ failure [41]. NF-κB transcriptional factors (TFs) are bound together in non-stimulated cells by inhibitory IκB proteins and then sequester into the cytoplasm. This activation results in the phosphorylation of IκB proteins, which are subsequently recognized by ubiquitinating enzymes. Then NF-κB TFs release and bind to IκB which prior IκB is degrading its proteasomal protein. Finally, NF-κB translocates to the nucleus and regulate the expression of target genes [42]. IKKα and IKKβ are protein kinases with a similar structure that conciliate phosphorylation of IκB proteins. They play a state of converging for many signal transduction pathways with the subsequent activation of NF-κB. Despite their similarity in the structure, IKKα and IKKβ have different functions relate to substrate specificity and types of regulation. IKKβ is important in the rapid activation of NF-κB through pro-inflammatory signaling cascades including those triggered by TNF-α and LPS [42]. In the current study, we found that LPS treatment alone and pretreatment with rHSC70 at 0.1 and 1 µg/ml led to the upregulation of IκBα and NF-κB p65. However, the expressions of IκBα and NF-κB p65 were attenuated following pretreatment with rHSC70 at 5 µg/mL. Similar to this, the EMSA of nuclear NF-κB binding and immunocytochemistry assay showed the attenuation of IκBα and NF-κB p65.

MMPs are a family of proteases whose expression is related to certain processes, such as development, physiology, and pathology and infections. Several MMP genes transcription can be stimulated by LPS, and abundant studies have been focusing on their signal transduction pathways which are responsible for their expression. This far, MMPs rely upon the activation of NF-κB and/or mitogen-activated protein kinases p38 and ERK1/2 [43]. LPS stimulation leads to the elevated expressions of MMPs, suggesting that it affects the pathogenesis of endotoxemia. The release of MMP-9 has been reported in healthy human volunteers after injections of bacterial LPS [44]. Respectively, a study has been reported that in patients with gram-negative bacteria, the severity of sepsis depends on the increasing levels of pro-MMP-9, pro-MMP-2, and the activated forms of MMP-9 [45]. However, another clinical study in patients who suffer from septic shock demonstrated that higher MMP-9 level was found in patients who suffocated from severe sepsis compared to the survivors and healthy controls [46]. In the current study, we found that LPS treatment alone and pretreatment with rHSC70 at 0.1 and 1 µg/mL caused upregulation of MMP-2 and MMP-9. On the contrary, these upregulations were attenuated in pretreatment with rHSC70 at 5 µg/mL. In addition, ERK1/2, JNK, p38 and Akt were upregulated following LPS treatment alone and pretreatment with rHSC70 at 0.1, 1 and 5 µg/mL, whereas the LPS-induced upregulation of ERK1/2, JNK, p38, and Akt were attenuated in a dose-dependent manner.

## 4. Materials and Methods 

### 4.1. Chemicals and Reagents

Enzo Life Sciences (Farmingdale, NY, USA) provided recombinant bovine HSC70, a low-endotoxin–containing preparation, containing less than 5.0 pg/µg protein (<0.05 EU/µg protein) of endotoxin, as measured by Limulus assay. Bacterial LPS (*Escherichia coli* serotype 026:B6, L8274) and antibodies against iNOS and β-actin were purchased from Sigma Aldrich Inc. (St. Louis, MO, USA). Cell Signaling Technology (Beverly, MA, USA) provided certain antibodies: phospho-ERK, phospho-Akt, Akt, phospho-IκBα, IκBα, and MMP-9. Meanwhile, Santa Cruz Biotech (Santa Cruz, CA, USA) supplied COX-2 and p38 antibodies. Upstate Biotechnology (Lake Placid, NY, USA) provided polyclonal antibodies against ERK and phospho-JNK. Monoclonal antibody against phospho-p38 was purchased from Abcam (Cambridge, MA, USA). Anti-p65 and anti-PARP were ordered from Millipore (Temecula, CA, USA), and anti-MMP-2 was from Thermo Scientific (Cheshire, UK). Dulbecco’s modified Eagle’s medium (DMEM), fetal bovine serum (FBS), streptomycin, penicillin, and all else tissue culture reagents were provided by GIBCO BRL Life Technologies (Grand Island, NY, USA).

### 4.2. Culture of Macrophages RAW264.7

The murine macrophage-like RAW264.7 cells were cultured in DMEM containing 10% fetal bovine serum, antibiotics and 1% glutamine at 37 °C in a humidified 5% CO_2_ incubator. All experiments were performed with exponentially growing cells.

### 4.3. Nitric Oxide Assay

The Griess assay was used to measure nitric oxide. Griess reagent (1% sulfanilamide and 0.1% *N*-(1-naphthyl) ethylenediamide in 5% phosphoric acid) was used to detect nitrite concentrations. An equal volume of Griess reagent mixed with cell culture supernatant and then incubated at room temperature for 10 min. The 540 nm (OD_540_) absorbance was measured. 

### 4.4. Measurement of TNF-α and IL-6 Levels

RAW264.7 cells (105 cells/mL) were seeded in 24-well plates. The cells were pretreated with various concentrations of HSC70 for 5 min, followed by incubation with 0.1 µg/mL LPS for 24 h. Subsequently, the media of cells were collected and then enzyme-linked immunosorbent assay (ELISA) was used to measure the levels of TNF-α and IL-6. ELISA assay was followed as manufacturer’s protocol (Pierce Biotechnology, Rockford, IL, USA).

### 4.5. Cytosolic and Nuclear Protein Extracts Preparation

NE-PER Nuclear and Cytoplasmic Extraction kit (Pierce Biotechnology) was used to separate and to prepare cytoplasmic (CE) and nuclear extracts (NE). The protocol was based on manufacturer's instructions. All of the fractionated protein solutions were stored at −80 °C until analysis.

### 4.6. Western Blot Analysis

RAW264.7 cells were treated by HSC70 with dosage and time period as defined. The reactions were terminated by twice washes with cold PBS. The cells were then harvested and western blot analysis were based previously studies [47,48].

### 4.7. Electrophoretic Mobility Shift Assay (EMSA)

The EMSA assay was based on a prior study [47]. Nuclear extracts were equipped and incubated with biotin-labeled NF-κB consensus oligonucleotides in reaction buffer for 20 min at room temperature. The evaluation of NF-κB binding to DNA specificity was using a double-stranded mutated oligonucleotide. The specificity was also determined through competition with unlabeled oligonucleotide.

### 4.8. Immunocytochemistry 

RAW264.7 cells were conducted by HSC70 with dosage and time period as determined. The cells were fixed with 10% formaldehyde for 30 min at 4 °C. After washing with PBS, the cells underwent mouse anti NF-κB overnight at 4 °C. Following FITC-conjugated secondary antibody incubation, the cells were visualized with an Olympus Fluoview FV1000 instrument (Olympus Optical Co., Tokyo, Japan). The cells nuclei were stained with DAPI.

### 4.9. Gelatin Zymography

Gelatin zymography on pre-made 8% polyacrylamide gels containing 0.1% gelatin was used to detect the activities of MMP-2 and MMP-9. The protocols followed a prior study [48]. After electrophoresis, the gel was removed and incubated in 1× zymogram renaturing buffer for 30 min at room temperature. The gel was equilibrated for 30 min with 1× zymogram developing buffer and then incubated with fresh 1× zymogram developing buffer overnight. The staining to detect the band were using a solution containing 0.1% Coomassie R-250 in 40% ethanol and 10% acetic acid. And then it continued to a de-stained process by embedding in a solution containing 10% ethanol and 7.5% acetic acid for 2 h at room temperature. The images were visualized in the UVP Biochemi EC3 imaging system (UVP, LLC, Upland, CA, USA).

### 4.10. Statistical Analysis

Data were expressed as a mean ± standard error of the mean (SEM). Statistical differences were estimated by one-way analysis of variance (ANOVA) followed by Dunnett’s test. A value of *p* < 0.05 was considered significant.

## 5. Conclusions

Our findings provided a novel evidence suggesting that p38 MAPK and ERK1/2 are involve in the pathways of HSC70-induced macrophages stimulated by LPS (endotoxin). This study provides possible explanations for the immunogenicity of HSC70. The prophylactic action of exogenous HSC70 allows the induction of professional APCs like macrophages to stimulate innate immune responses like the emergence of phagocytes in an earlier stage of inflammation. These properties provide the administration of HSC70 as an infection immunotherapy in novel ways. The next goal should be the identification of LPS stimulation followed by HSC70 administration and possible mechanisms that result in the immune response. Further studies are essential to achieve the beneficial application of exogenous HSC70 in immunotherapy.
